# The first mitochondrial genome of *Sepsis monostigma* (Diptera: Sepsidae)

**DOI:** 10.1080/23802359.2021.1959431

**Published:** 2021-07-29

**Authors:** Pan Yang, Jianfeng Wang

**Affiliations:** Liaoning Key Laboratory of Urban Integrated Pest Management and Ecological Security, College of Life Science and Bioengineering, Shenyang University, Shenyang, China

**Keywords:** Sepsidae, *Sepsis monostigma*, mitochondrial genome, phylogeny

## Abstract

The sepsid fly *Sepsis monostigma* belongs to the genus *Sepsis* of Sepsidae. We sequenced and annotated the mitogenome of *S. monostigma* which as the first representative of genus *Sepsis* with nearly complete mitochondrial data. This mitogenome is 14,887bp long, which contains of 22 transfer RNA genes, 13 protein coding genes (PCGs) and 2 ribosomal RNA genes and a part of the AT control region. ML phylogenetic outcome strongly supported the monophyly of Sepsidae, and the family Sepsidae is more close to the family Heleomyzidae. It also indicated that the genus *Sepsis* is the sister group to *Nemopoda*, and the genus *Archisepsis* is the sister group to *Microsepsis*.

Sepsidae is a global distributed fly family with more than 320 described species (Ozerov 2005), and it is a relatively small, morphologically and ecologically uniform family of the Sciomyzoiedea in the acalyptrate series of Diptera (Pont and Meier 2002). *Sepsis monostigma* (Thmpson,1869) is a rather important model-organisms insect and a sanitary fly of biological significance, which can perify the ecosystem (Li et al. 2015). The mitochondrial genome was often used for the research of species identification and phylogenetic relationships (Fu et al. 2016). Due to their tiny size, usually 2–12 mm, it is very difficult to extract enough genomic DNA. Hence, we sequenced the mitochondrial genome of *S*. *monostigma*, which is the first been reported in the Genus *Sepsis*. This study develop mitochondrial genome data of Sepsidae species, contributes to discussion on the evolutionary relationshios and provides theoretical basis for sexual seletion research.

*S. monostigma* (Voucher: Sepsidae001) was collected on 19 September 2020 at Bayi Park (latitude: 123.5236 N and longitude: 41.5243 E), Shenyang City, Liaoning Province, China. Each specimen was identified by entomologist according to taxonomic keys (Ang and Meier 2010). The voucher specimens and their DNA were deposited in the Natural History Museum of Shenyang University (JianfengWang, wangjf80@126.com) under the voucher number Sepsidae001.

The genomic DNA was extracted from the adult’s muscle tissues of the leg using the TIANamp Genomic DNA Kit (TIANGEN, Beijing, China), and sequenced under the next generation sequence technology with 22 primer pairs (Simon et al. 1994; Zhang et al. 2013; Zhao et al. 2013; Li et al. 2015). DNA fragments were sequenced on both strands by Applied Biosystems 3730 DNA Analyzer (Sangon Biotech Co. Ltd., Shanghai, China). Genome sequence was assembled by DNAman Version 6 software and was annotated by MITOS2 online software (http://mitos2.bioinf.uni-leipzig.de). Meanwhile, High-throughput sequencing was performed using Illumina Hiseq PE 150 Platform, using NOVOPlasty in de novo assembling. Almost the entire mitochondrial genome has been accurately obtained by two methods, except for the absence of the AT control region. This mitogenome of *S*. *monostigma* is 14,887 bp long, which contains of 22 transfer RNA genes, 13 protein coding genes (PCGs), 2 ribosomal RNA genes and a part of the AT control region, it was similar with related species reported before (Simon et al. 1994; Zhang et al. 2013; Zhao et al. 2013; Li et al. 2015; Chen et al. 2018; She et al. 2020).

The nucleotide composition of *S*. *monostigma* mitochondrial genome was 37.9% of A, 9.9% of G, 13.9% of C and 38.3% of T. The A + T content was 76.2% which conforms to the characteristics of AT preference like other flies. The codon ATG was the most popular start codon shared with the *COII*, *ATP6*, *COIII*, *NAD4*, *NAD4L*, *CytB*, and start codon ATT was shared with *NAD2*, *ATP8*, *NAD3*, *NAD5*, *NAD6*. Particularly, the *COI* begins with codon TCG, and the *NAD1* begins with codon TTG. The conservative stop codon TAA was shared with *NAD2*, *COI*, *COII*, *ATP8*, *ATP6*, *COIII*, *NAD4L*, *NAD6*, *NAD1*. Another common stop codon TAG was shared with *NAD3* and *CytB*. Particularly, the *NAD5* and *NAD4* stop with codon T. To make sure the authenticity of gene boundaries, all PCGs were compared with other muscid mitochondrial sequences under performed in MEGA 7.0 (Kumar et al. 2016).

Maximum Likelihood (ML) Phylogenetic analysis of four Sepsidae species, two Heleomyzidae species, three Drosophilidae speceis and four Muscidae species as the outgroup based on 13 PCGs by MEGA 7.0 software with 1000 bootstrap replicates (Kumar et al. 2016), and the topology is shown in Figure 1. According to the phylogenetic outcome, the outgroups four Muscidae species form a clade. Our results strongly supported the monophyly of Sepsidae, and the family Sepsidae is more close to the family Heleomyzidae. It also indicated that the genus *Sepsis* is the sister group to the genus *Nemopoda*, and the genus *Archisepsis* is the sister group to the genus *Microsepsis*. In addition, the mitochondrial genome sequences could also be useful in identifying Sepsidae species.

**Figure 1.**
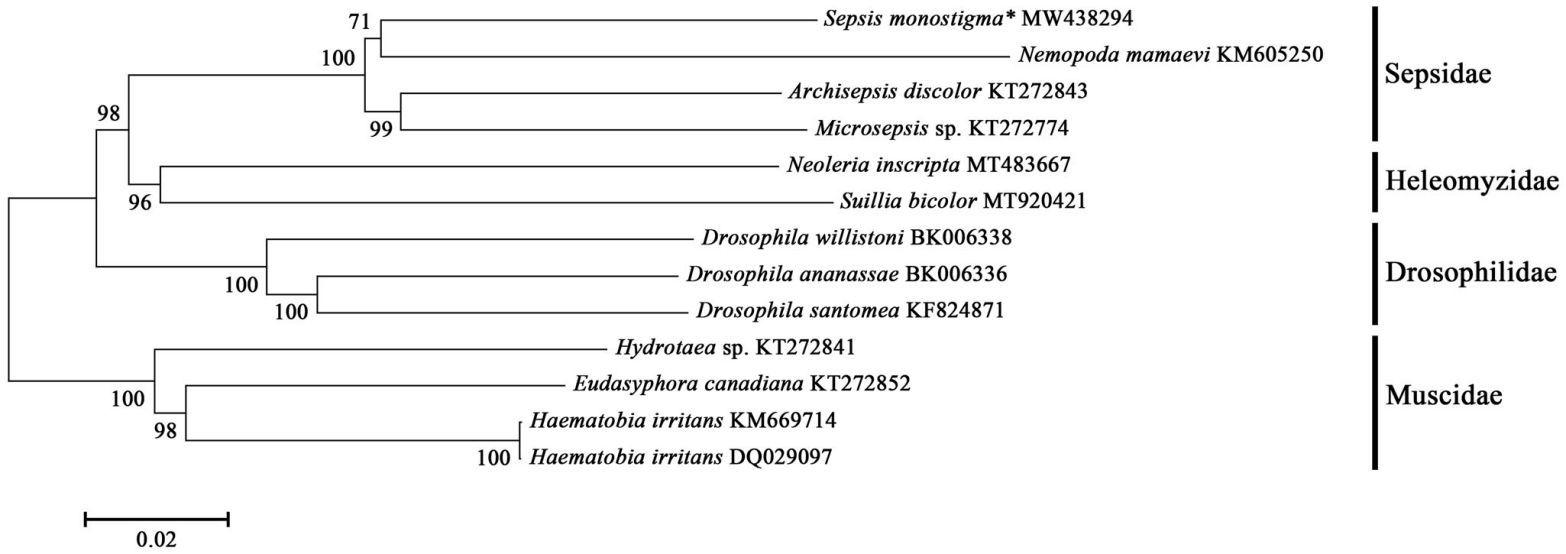
ML phylogenetic tree of thirteen species which consist of four Sepsidae species, two Heleomyzidae species, three Drosophilidae speceis and four Muscidae species as the outgroup using MEGA 7.0 software. The numbers in the nodes indicated the support values with 1000 bootstrap replicates. *indicates this study.

## Data Availability

The genome sequence data that support the findings of this study are openly available in GenBank of NCBI at (https://www.ncbi.nlm.nih.gov/) under the accession no. MW438294.
